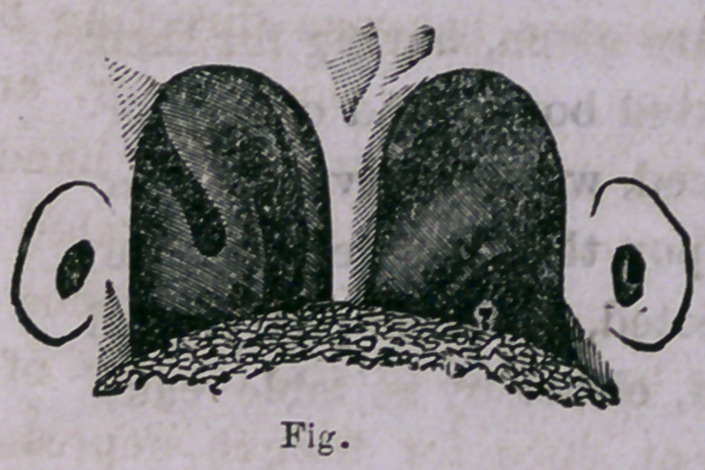# Practical Papers on Diseases of the Throat and Air Passages

**Published:** 1866-12

**Authors:** Edward B. Stevens

**Affiliations:** Professor of Materia Medica in the Miami Medical College of Cincinnati


### Miscellaneous.

### Practical Papers on Diseases of the Throat and Air Passages.

BY EDWARD B. STEVENS, M. D.,
Professor of Materia Medica in the Miami Medical College of Cincinnati.

*Rhinoscopy.*—Former papers have served to explain some general idea of the plan of procedure, and the leading advantages to be obtained in the diagnosis of diseases of the larynx by *laryngoscopy.* I have desired to give such clear but brief description of the operation, together with wood cut illustrations, that the reader of this journal may for himself enter upon the use of the laryngeal mirror without special instruction. Hereafter we propose to give a series of cases illustrative of practical diagnosis and therapeutics; and connected with these papers, my friend, Dr. Bruhl, has in course of preparation additional contributions bearing upon the same topics. Before commencing these, however, it is our plan at present to explain and illustrate what is meant by *Rhinoscopy.*

Hitherto the inspection of the posterior nares has been for practitioners quite as much a *terra incognita* as the internal space of the larnyx. We now propose to study the condition of that region by the same plan of mechanism as is pursued for *laryngoscopic* examinations, that is to say, the principle is precisely the same, the details being but slightly modified.

In rhinoscopy, as in laryngoscopy, a laryngeal mirror is employed, but for our present purpose, a *small mirror* will almost always be found more readily adapted: a slightly different angle of attachment between the mirror and handle will be necessary, but this will naturally occur to the manipulator.

The illumination is made in the same manner; the strong direct solar rays being satisfactory, or a good arg and lamp reflected from a Czermak mirror, or a strong cone of light through a Tobold condenser. There will be found, however, a necessity for a more brilliant illumination in rhinoscopy than in laryngoscopy.

An additional contrivance is necessary in this operation, a fenestrated hook with which to hold up, out of the range of vision, the pendant uvula; a little trick also which to some extent serves to expand the opening to the posterior nares and thus still further admit light, and of course facilitate inspection.

Many cases will require the use of a tongue depressor, and it at once becomes manifest that the operator between directing his light, controlling the uvula and velum, holding his mirror, and depressing the tongue, will have employment for all his hands. To obviate this difficulty in part, the patient may be instructed in the use of the depressor, or as is suggested in a contrivance of Voltolini, a shield attachment may be made to the handle of a mirror so adjustable that it serves at once for tongue depressor and rhinoscopic mirror.

With these explanations the reader will be ready to understand the following wood cut, illustrating the mode of procedure, which we copy from *Bennett*—Wm. Wood & Co.'s last edition:

Fig. 1

A general view is afforded of all the parts directly or indirectly concerned in this inspection. A section of the cervical vertebræ, the epiglottis and larynx, the naso-pharyngeal structures and cavity, and the rhinoscopic mirror in position.

It will be observed that the uvula is held up by the hook, at the same time that two positions, at z and z, of the mirror are indicated.

To conduct this inspection satisfactorily, requires more patient cultivation of tact than for laryngoscopy, but the tact is similar, and obtained by a study of like arts, such as the laryngoscopic operator learns to render available.

Fig.

Fig. 2 gives a view of the posterior nares as seen in the rhinoscopic mirror; there is seen the posterior orifices of the nasal fossa, the turbinated bones, and on the extreme border of either side the orifices of the eustachian tubes. This illustration is taken from the original views given by Czermak.

A few words in brief memoranda of the applications of this part of our diagnostic art will suffice for our present purpose, and indicate sufficiently its importance to the practitioner.

In the cut above, (Fig. 2,) it is observed how easily the orifices of the eustachian tubes are brought into range of inspection; the usual plan for reaching these orifices especially pursued for purpose of catheterization in aural surgery, is by means of a catheter, introduced along the floor of the nasal cavity, a maneuvre which requires quite as much dexterity as any part of the art of rhinoscopy, and it is very clear that a careful rhinoscopic observation will not only facilitate this delicate operation when necessary to be performed, but will enable the operator to introduce a catheter with less danger of violence to the structures.

General symptoms will serve to indicate for us states of ulceration, catarrhal inflammation, and other pathological changes, but the inspection hereby afforded, detects the exact character of these conditions and their exact locality; thus indicating both the kind and location of treatment demanded.

The more common morbid conditions discovered by the rhinoscope, are these catarrhal inflammations, ulcerations and destruction of parts, and morbid growths.

Czermak gives two cases of deafness in which the rhinoscope revealed redness and œdema of the naso-pharyngeal surface, especially involving the tissues about the orifices of the eustachian tubes. Other operators have discovered matters actually plugging up the orifice, and thus producing deafness.

In Semeleder's* interesting little volume, quite a number of cases of mucus and polypoid growths are given, having for their attachment various points of the turbinated bones, and other portions of the posterior nasal opening. Indeed, we may have like morbid growths upon this entire surface as upon the laryngeal surface; fortunately for the most part, when detected, the naso-pharyngeal growths will be the more easily removed, either by suitable caustics or by the polypus forceps and scissors.

Semeleder, Voltolini and Czermak, relate very many cases of ulcëration, ozœna, morbid growths, etc., revealed by this mode of

---

* Rhinoscopy and Laryngoscopy; by Dr. F. Semeleder: Wm. Wood & Co., New York, 1866.

examination. The treatment will, of course, not be particularly different from that demanded for like diseased conditions in other localities, but the treatment is pursued with definiteness and precision. Many of the ulcers discovered by these operators, evidently from their history, had a syphilitic origin and yielded to the proper constitutional remedies.

For the local medication of these surfaces, and the application of remedies, much the same instruments are employed, as in operations and applications to the laryngeal surface. Hereafter we may contribute something more in detail of cases, further illustrating this field of operation.—*Cincinnati Lancet and Observer.*

## Figures and Tables

**Fig. 1 f1:**
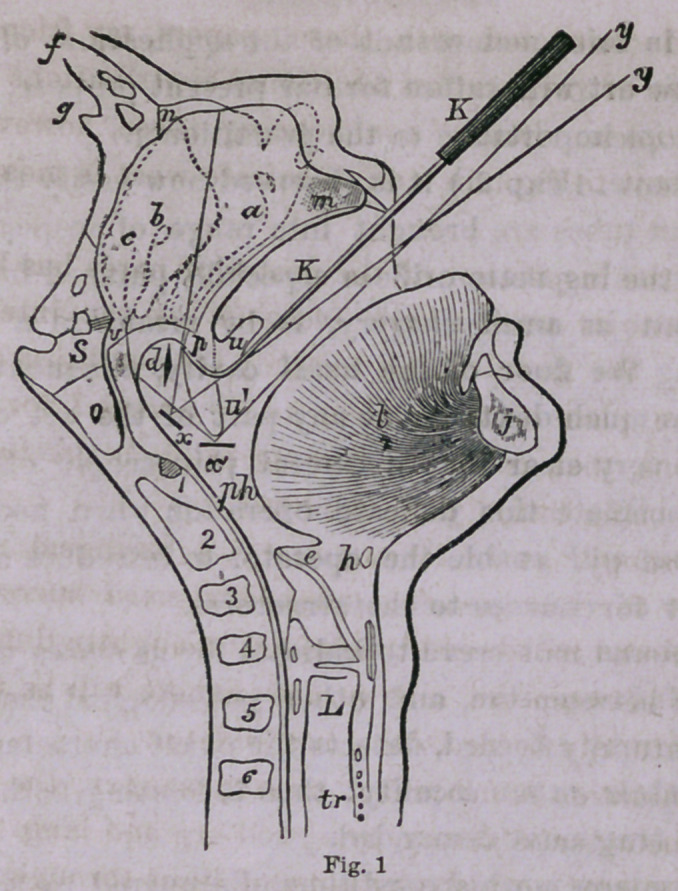


**Fig. f2:**